# Supplementary material for: Splice-Junction-Based Mapping of Alternative Isoforms in the Human Proteome
Source: Cell Rep. Author manuscript; Available in PMC 2020 Jan 15. (PMC6961840; doi:10.1016/j.celrep.2019.11.026)
Supplement: 2 [file NIHMS1546469-supplement-2.zip › DF1/PXD010154/Ovary/Ovary_11_USP40_PPQSLDGGEEAPRQAFTNYSTVSNNQYGK.pdf]

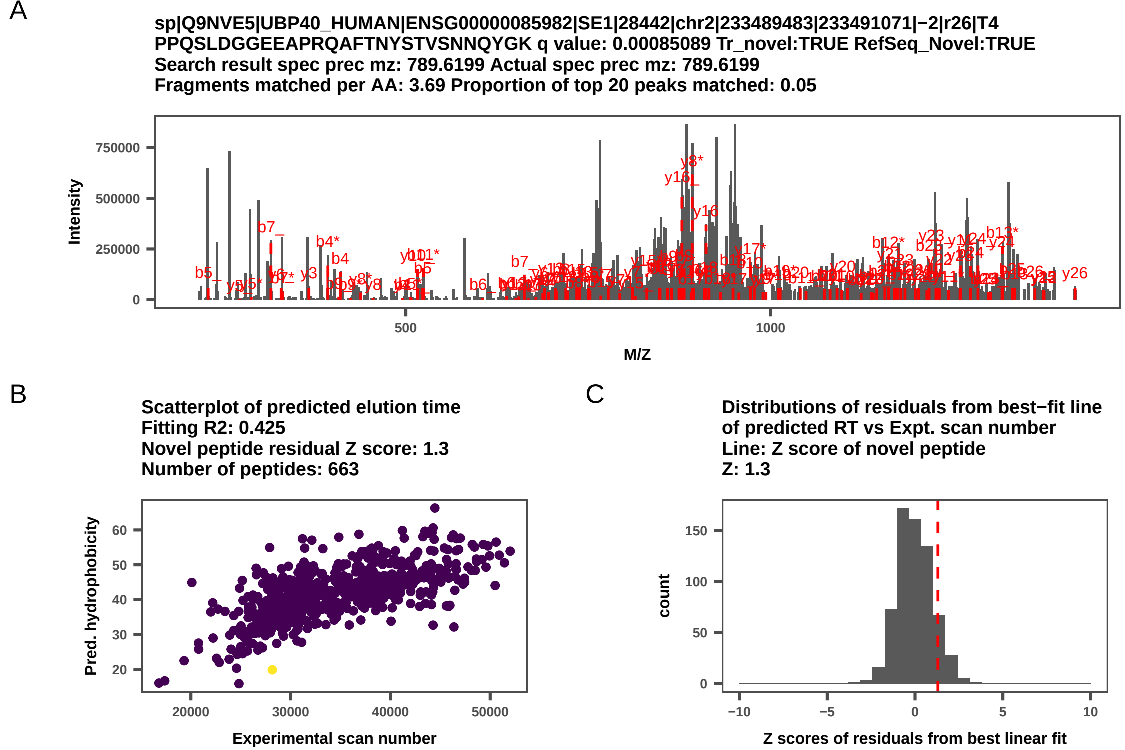
A
sp|Q9NVE5|UBP40_HUMAN|ENSG00000085982|SE1|28442|chr2|233489483|233491071|−2|r26|T4
PPQSLDGGEEAPRQAFTNYSTVSNNQYGK q value: 0.00085089 Tr_novel:TRUE RefSeq_Novel:TRUE
Search result spec prec mz: 789.6199 Actual spec prec mz: 789.6199
Fragments matched per AA: 3.69 Proportion of top 20 peaks matched: 0.05
Intensity
750000
500000
250000
0
500
1000
M/Z
B
Scatterplot of predicted elution time
Fitting R2: 0.425
Novel peptide residual Z score: 1.3
Number of peptides: 663
Pred. hydrophobicity
60
50
40
30
20
20000
30000
40000
50000
Experimental scan number
C
Distributions of residuals from best−fit line
of predicted RT vs Expt. scan number
Line: Z score of novel peptide
Z: 1.3
count
150
100
50
0
−10
−5
0
5
10
Z scores of residuals from best linear fit
